# Antimicrobial Potential of Six Plant Essential Oils Against *Pseudomonas syringae* pv. *actinidiae*: *In Vitro* Activity and *In Planta* Efficacy Do Not Always Align

**DOI:** 10.3390/plants14243825

**Published:** 2025-12-16

**Authors:** Marta Nunes da Silva, Miguel G. Santos, Marta W. Vasconcelos, Susana M. P. Carvalho

**Affiliations:** 1 GreenUPorto—Sustainable Agrifood Production Research Centre/Inov4Agro, Departamento de Geociências, Ambiente e Ordenamento do Território (DGAOT), Faculty of Sciences, University of Porto, Rua da Agrária 747, 4485-646 Vila do Conde, Portugal; mansilva@ucp.pt (M.N.d.S.); mgsantos@fc.up.pt (M.G.S.); 2 CBQF—Centro de Biotecnologia e Química Fina—Laboratório Associado, Escola Superior de Biotecnologia, Universidade Católica Portuguesa, Rua Diogo Botelho 1327, 4169-005 Porto, Portugal; mvasconcelos@ucp.pt

**Keywords:** antimicrobial activity, crop protection, kiwifruit bacterial canker, *Pseudomonas syringae* pv. *actinidifoliorum*

## Abstract

Plant essential oils (EOs) are attracting interest as ecofriendly alternatives to antibiotics and copper-based control of kiwifruit bacterial canker (KBC), caused by *Pseudomonas syringae* pv. *actinidiae* (*Psa*). This study chemically profiled six EOs (anise, basil, cardamom, cumin, fennel, and laurel) and evaluated their antimicrobial activity both *in vitro* and *in planta*. The *in vitro* assay targeted four strains, two of *Psa* and two of the low-virulent *P. syringae* pv. *actinidifoliorum* (*Pfm*), whereas the *in planta* assay focused on the highly virulent *Psa*7286 strain, assessed under preventive and curative application regimes (i.e., 14 days pre- or post-inoculation, respectively). Cumin, with cuminaldehyde as its major component (48%), was the most effective EO in vitro, significantly inhibiting growth at 5–10% concentration, whereas anise, rich in anethole (89%), was consistently the least effective one. However, the *in planta* application of the EOs produced antimicrobial effects that differed markedly from *in vitro* results and showed strong dependence on the timing of application. Preventive treatment significantly reduced *Psa* endophytic populations in basil (70%), anise (54%), laurel (42%), and cumin (35%) compared to untreated plants. In contrast, when the EOs were applied post-inoculation (curative treatment), a significant decrease in *Psa* colonization was observed in laurel (81%), cardamon (70%), cumin (31%) and fennel (29%). Although plant EOs are gaining momentum in the control of *Psa* and other diseases, translation from *in vitro* to *in planta* efficacy is not direct and is strongly timing-dependent, which underscores the need to perform validation trials *in planta* and to fine-tune application schedules for the integrated management of KBC.

## 1. Introduction

Kiwifruit bacterial canker, caused by *Pseudomonas syringae* pv. *actinidiae* (*Psa*), is a major constraint to plant growth and productivity, driving substantial economic losses and demanding rigorous orchard management [1]. This pathogen is now reported from all major kiwifruit-producing regions and can infect virtually all *Actinidia* species, including cultivated *A. chinensis* and *A. arguta* [2]. By contrast, *P*. *syringae* pv. *actinidifoliorum* (*Pfm*) is less virulent; although it does not typically cause severe yield losses, it compromises plant fitness through necrotic leaf spots [3,4]. Control of both Gram-negative pathovars is important for crop performance, yet research attention has focused predominantly on *Psa* due to its global impact on kiwifruit health [5]. These efforts have delivered several candidate control strategies, including plant defense inducers and microbial biological control agents, at varying levels of technological maturity, with few products currently commercialized [6]. In practice, management has relied largely on copper-based compounds and, where permitted, antibiotics such as streptomycin. However, their continued use is increasingly discouraged due to limited and inconsistent efficacy and to environmental and human health concerns [7,8,9,10]. Despite incremental advances, robust and reliable control of *Psa* remains elusive, and antibacterial options for *Pfm* have received comparatively little attention [11]. In fact, closely related nonpathogenic or mildly virulent populations, such as *Pfm*, can act as reservoirs of virulence factors, enabling the emergence of more aggressive variants [5]. There is, therefore, a clear need to broaden the portfolio of safe and effective tools for kiwifruit disease management. In particular, molecules that prevent infection and/or lower endophytic bacterial loads in already infected plants are a priority for integrated programs [6].

Plant essential oils (EO) are promising candidates because they combine direct antimicrobial activity with the capacity to elicit plant defense responses and may also enhance copper efficacy when used in nanoformulations [6,12,13,14]. These complex mixtures can include substances from tens of chemical groups, with one or a few bioactive constituents often accounting for most of the composition [15,16,17]. Reported antibacterial mechanisms include disruption of the cell envelope that increases membrane permeability and leads to leakage of cellular contents, as well as interference with motility, the type III secretion system, and quorum sensing [18,19,20]. Many essential oils and their constituents are active at low concentrations, a practical advantage since phytotoxicity and poor water miscibility often necessitate low-concentration emulsions stabilized with surfactants [21,22]. However, *in planta* evaluations of EOs have focused mainly on fungal and oomycete diseases, while bacterial targets are less studied. Seed and soil treatments with plant EOs have reduced disease caused by diverse fungi in several horticultural crops, although effective concentrations, carriers, and timings vary [23,24,25,26,27,28]. For example, seed priming with EOs from cumin, basil, and geranium at 4% reduced root rot in cumin caused by *Fusarium* spp. [23], while seed priming with oregano EO at 12% also lowered the severity of *Sclerotinia sclerotiorum* in lima bean [28]. In potted tomato seedlings, oregano and clove EOs at concentrations up to 0.01% significantly decreased symptoms caused by *Botrytis cinerea* and *Ralstonia solanacearum*, respectively [24,25]. Likewise, clove EO emulsions at up to 10% limited disease caused by *Fusarium oxysporum* f. sp. *lycopersici* in potted tomato seedlings [26]. In greenhouse trials, a soil drench with lemon EO at 50 mL per plant reduced symptom severity caused by *Phytophthora* sp. on pepper, cucumber, and melon relative to untreated controls [27]. More recently, garlic and cinnamon essential oils have been encapsulated in silver nanoparticles, which enhanced their fungicidal activity against Botrytis cinerea, a major pathogen of horticultural crops [29]. The nanoformulated oils inhibited mycelial growth and conidial germination and caused cell wall disruption and deformed hyphae. In addition, chitosan nanoparticles loaded with lemon or spinach seed essential oils showed strong antifungal activity against *Penicillium expansum* (blue mold of apple) and *Podosphaera fusca* (powdery mildew of cucumber), while inducing defense-related and antioxidant enzymes, including polyphenol oxidase, peroxidase and phenylalanine ammonia lyase, without compromising fruit quality [30,31].

Most work on *Psa* has evaluated the antibacterial activity of PEOs and plant extracts *in vitro* [15,16,17,19], whereas *in planta* efficacy against *Psa* remains limited [13,20,32]. In kiwifruit, foliar applications of EOs from savory, thyme, and pennyroyal have shown inconsistent outcomes across studies [13,32]. Under greenhouse conditions, applying savory and thyme EOs 24 h before *Psa* inoculation, followed by nine further applications over 15 weeks, reduced both the number of diseased leaves and the affected leaf area. In contrast, in micropropagated plants treated 3 days before inoculation, winter savory at 5 mg.mL^−1^ produced negligible inhibition relative to pennyroyal [13]. Also, cinnamon EO-based emulsion markedly decreased disease frequency and severity, although protection was weaker when cinnamon was combined with oregano and when treatment was applied 24 h after inoculation rather than 24 h before [20]. Under field conditions, cinnamon EO applied at bloom and then at three monthly intervals was more effective than cinnamon or a cinnamon plus oregano mixture in lowering the disease index for up to four months after treatment. However, when oregano was combined with cinnamon, the mixture performed worse than cinnamon alone, indicating that oregano can diminish the protective effect of cinnamon [20]. Together with the weaker protection observed when cinnamon was applied after inoculation rather than before, these results point to complex, non-additive interactions among EOs and emphasize that mixture composition and application timing critically shape outcomes. In addition, variability across studies likely arises from differences in EO chemistry, the genotypes of both bacteria and host plants, and the formulation, concentration, application method and timing, and growth conditions used. These sources of heterogeneity underscore the need for standardized comparative trials that explicitly contrast preventive and curative schedules.

In this context, chemical profiling is essential to interpret bioassays, identify candidate bioactive constituents, and improve comparability across studies; however, many reports do not include full composition data. In several cases, chemical analyses of PEOs with stronger anti-*Psa* activity identified piperitenone oxide in apple mint, pulegone and menthone in pennyroyal, borneol in rosemary, caryophyllene in sage, camphene and cinnamyl acetate in laurel, and terpinene-4-ol in tea tree as the most abundant constituents [13,16]. Individual compounds such as carvacrol and juglone also showed strong inhibition of *Psa* by disrupting cell membrane permeability and integrity [33,34], and cinnamaldehyde, eugenol, estragole, and methyl-eugenol also inhibited in vitro, with efficacy varying across *Psa* strains [15]. Yet, focusing on single constituents does not fully explain whole-oil performance because constituent interactions can be synergistic or antagonistic. For example, eugenol, estragole, and methyl-eugenol exhibited high inhibitory activity, but extracts of West Indian Bay tree and Jamaica pepper, both rich in these molecules, were only as effective as Chinese cinnamon, where these compounds were not detected [15]. Similarly, although bergamot oil contained more thymol than a closely related wild species (59–64% versus 38–42%), their anti-*Psa* activities were comparable, likely due to carvacrol present in wild bergamot (at about 3.9%) [17]. These patterns underline that overall antimicrobial potential reflects the full chemical matrix rather than any single dominant component. In addition, the potential of plant EOs against *Pfm* remains largely unexplored. Although this pathovar is less destructive than *Psa*, its management is relevant in orchards where multiple *Pseudomonas* pathovars may co-occur. Moreover, despite the recent withdrawal of the European Commission’s proposal to halve pesticide use by 2030 under the “Farm to Fork Strategy” due to lack of political consensus, the need to develop sustainable alternatives remains urgent. In this regard, incorporating plant EOs as both preventive and curative control strategies against KBC will contribute to achieving this objective. As such, this study tests the hypothesis that EOs effective *in vitro* against *Psa* and *Pfm* strains will also exhibit activity *in planta* against *Psa*, with their performance being conditioned by the application timing. Six widely used spice and herb oils—anise, cumin, fennel, basil, laurel, and cardamom—were therefore evaluated to: (i) quantify *in vitro* antibacterial activity against both pathovars; (ii) compare the sensitivity of strains differing in virulence; (iii) validate preventive and post-infection efficacy *in planta* against *Psa*7286; and (iv) characterize chemical composition to relate constituents to antimicrobial performance and to identify candidates for future management tools.

## 2. Results

### 2.1. In Vitro and In Planta Antimicrobial Activity Against Psa and Pfm

**Figure 1:**
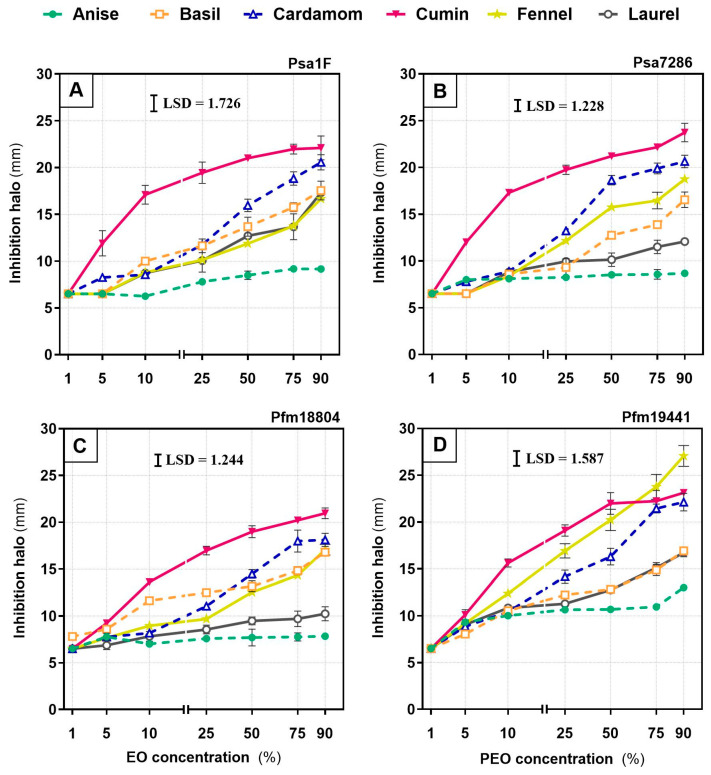
Inhibition zone diameter (mm) of *Pseudomonas syringae* pv. *actinidiae* (*Psa*; strains 1F—(**A**), and 7286—(**B**)) and *P. syringae* pv. *actinidifoliorum* (*Pfm*; strains 18804—(**C**), and 19441—(**D**)) measured after 48 h exposure to six essential oils: anise, basil, cardamom, cumin, fennel, and laurel. Oils were tested at 1, 5, 10, 25, 50, 75 and 90% *v*/*v*. Each symbol is the mean of three biological replicates, and error bars indicate the standard error of the mean. The least significant difference (LSD) at *p* < 0.05 is shown in each panel.

Across the four strains and six essential oils, *in vitro* inhibition increased with oil concentration, with strong concentration–response relationships (R^2^ = 0.67–0.99). The exception was anise against *Psa*7286 and Pfm18804, which showed weaker fits (R^2^ = 0.50 and 0.42, respectively; Supplementary Materials Table S1). Overall, cumin was the most effective in vitro, producing large inhibition halos at comparatively low concentrations (Figure 1).

Cumin generally yielded inhibition halos from 9.2 ± 0.6 mm at 5% to 23.7 ± 1.0 mm at 90% across all strains, with fennel significantly outperforming cumin against *Pfm*19441 at 90% (reaching 27.1 ± 1.1 mm). In contrast, anise was consistently the least active EO, with the smallest halos even at 90%, ranging from 8.7 ± 0.4 mm for *Psa*7286 to 13.0 ± 0.2 mm for *Pfm*19441. Basil, cardamom, and laurel showed intermediate activity. At 5%, their halos reached up to 8.6 ± 0.2 mm, 8.8 ± 0.4 mm, and 9.1 ± 0.2 mm, respectively, and at 90% they reached 17.5 ± 1.0 mm, 22.1 ± 0.9 mm, and 17.3 ± 0.3 mm, respectively. However, some strain-specific differences were evident, including within pathovars. For example, at 90%, among *Psa* strains, *Psa*1F was more sensitive to laurel than *Psa*7286 (*p* < 0.0001) but less sensitive to fennel (*p* < 0.01), and among *Pfm* strains, *Pfm*19441 was more sensitive than *Pfm*18804 to anise (*p* < 0.0001), cardamom and cumin (*p* < 0.05), fennel and laurel (*p* < 0.01).

**Figure 2:**
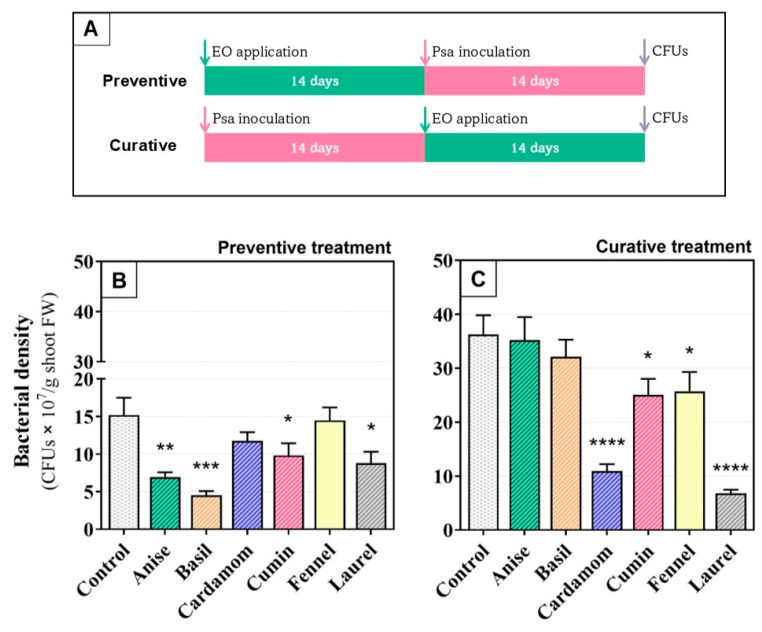
(**A**) In planta application of six plant essential oils at 0.1% (*v*/*v*), 14 days pre-inoculation (preventive treatment) or 14 days post-inoculation (curative treatment) and the respective (**B**,**C**) endophytic population of *Pseudomonas syringae* pv. *actinidiae* in shoots of *Actinidia chinensis* var. *deliciosa* ‘Tomuri’ expressed as CFU·g^−1^ fresh weight. Control plants were inoculated but received no essential oil treatment. Bars represent the means of three biological replicates, and error bars indicate the standard error of the means. Different letters denote significant differences among treatments within each panel (ANOVA with Fisher’s LSD, *p* < 0.05). Significance levels are indicated as follows: ****, *p* < 0.0001; ***, *p* < 0.001; **, *p* < 0.01; *, *p* < 0.05.

Concerning the *in planta* trials, in untreated inoculated plants (control), the endophytic population of *Psa* increased over time, reaching 15 ± 4.0 × 10^7^ colony-forming units per gram (CFU·g^−1^) of shoot tissue at 14 days post inoculation (dpi) and 36 ± 6.2 × 10^7^ CFU·g^−1^ at 28 dpi (Figure 2B and Figure 2C, respectively).

Preventive application of EOs significantly reduced *Psa* colonization for basil (70%), anise (54%), laurel (42%), and cumin (35%), relative to the untreated control, whereas cardamom and fennel produced no significant change (Figure 2B). In contrast, when EOs were applied as curative treatment against *Psa* basil and anise had no significant effect, whereas laurel, cardamom, cumin, and fennel lowered *Psa* loads by 81, 70, 29, and 31%, respectively (Figure 2C). Overall, anise and basil only worked out as preventive treatments, whereas cardamom and fennel were only effective as curative treatments. Interestingly, cumin and laurel had broader action, with significant efficacy in both preventive and curative application regimes.

### 2.2. Chemical Characterization of the EOs

Chemical profiling revealed differing levels of complexity among the studied EOs (Table 1, Supplementary Materials). Anise, basil and cardamom contained the largest number of identified constituents, from 23 to 24 compounds, whereas fennel, cumin and laurel had 18 to 20. A closer look at relative abundances highlights distinct chemical signatures (Table 1).

Anise showed the highest levels of anethole (89%), while basil was characterized by very high estragole (93%). Cardamom was rich in β-himachalene (78%), with lower amounts of D-limonene (7.4%), and linalyl acetate (3.8%). Cumin showed elevated cuminaldehyde (48%), β-cymene (19%), and 3-carene (14%). Fennel was dominated by 1,8-cineole (69%; also known as eucalyptol), with β-himachalene (17%), bornyl acetate (1.7%), terpinen-4-ol and γ-terpinene (both at 1.0%). Laurel contained substantial anethole (78%), fenchone (9.1%), and estragole (6.7%). Despite these differences, all EOs shared four compounds: estragole (0.44% to 93%), linalyl acetate (0.02% to 3.8%), and terpinen-4-ol (0.02% to 1.0%) and ρ-cymene (0.01% to 0.89%). On the other hand, several constituents were exclusive to a single EO. For example, longifolene, thunbergol and γ-himalachene were only detected in anise (at 3.8, 1.5, and 0.55%, respectively), β-isocumene and nerol acetate in cardamom (at 1.2 and 1.1%, respectively), and chrysanthenol and phellandral were detected only in cumin (at 9.2 and 0.69%, respectively).

## 3. Discussion

Kiwifruit bacterial canker caused by *Psa* requires a diversified toolkit of sustainable control molecules that can limit endophytic colonization and help manage the emergence of chemical resistance [9,10]. Although *Pfm* is less virulent and typically poses limited direct economic risk, coinfection could weaken plant fitness and potentially exacerbate the impact of *Psa* where both occur [35]. Within this context, the present study contributes to identify sustainable options by expanding our knowledge on the list of EOs with activity against *Psa* and, for the first time, exploring potential inhibitory effects against *Pfm*.

In the In vitro assays, all tested essential oils significantly inhibited both pathovars in a predominantly concentration-dependent manner. Nevertheless, cumin was consistently the most active EO, while anise showed the weakest activity. Overall, several oils showed steep increases in activity at low concentrations (≤10%), which is encouraging for practical use since low doses facilitate emulsification and reduce phytotoxic risk [22]. In this context, microencapsulation and other nanotechnology-based delivery systems could improve stability and provide controlled release, enabling equivalent or greater protective effects at even lower application rates and thereby reducing the total oil required [36,37]. among the oils with intermediate *in vitro* effects, fennel was the most effective against *Pfm*19441 at 75% and 90% concentrations. Nonetheless, for *Pfm*18804 and at all other concentrations, cumin remained the most active EO. Such variation, within and between pathovars, aligns with previous reports and argues for tailoring oil selection to pathogen lineage or using rational combinations that target multiple mechanisms simultaneously [15]. Indeed, synergistic activity from subinhibitory combinations has been shown for rosemary, tea tree, and apple mint against *Psa* [16] and, therefore, systematic testing across strain panels will be needed to map predictable response patterns and anticipate limitations. Our results substantiate prior evidence that basil, cumin, fennel, and laurel can inhibit *Psa in vitro* [16,19] and newly document activity for anise and cardamom. Earlier reports of no cardamom effect against *Psa* may reflect differences in chemotype or methodology, as composition was not disclosed [19]. Here, cardamom shared some constituents with published profiles, such as D-limonene and linalyl acetate, while being rich in β-himachalene, a feature also linked to antibacterial and antioxidant activity in Atlas cedar oil [38,39]. Conversely, discrepant findings for rosemary across studies illustrate how strain choice, plant chemotype, extraction method, concentration, and growth conditions can shift outcomes, underscoring the value of meta-analyses to resolve patterns and optimize use conditions [40,41,42]. Crucially, this study extends evidence from plates to plants. We show that cumin, fennel, basil, cardamom, laurel, and anise can reduce endophytic *Psa* loads *in planta*, but efficacy depends on timing. Preventive applications favored basil and anise, curative applications favored laurel and cardamom, while cumin performed in both windows, albeit with different magnitudes.

The mismatch between *in vitro* activity and *in planta* protection in some cases can be partly explained by additional barriers and host responses that govern outcomes in living tissues. At plant level the EOs can trigger defense mechanisms (e.g., improved antioxidant response and phytohormone modulation) that go beyond a direct pathogen growth inhibition [13,43]. Composition activity relationships help interpret these patterns but are not strictly additive. For instance, oils active against *Psa* often contain piperitenone oxide, pulegone, menthone, borneol, caryophyllene, camphene, cinnamyl acetate, and terpinene-4-ol, and single compounds such as carvacrol and juglone can disrupt membranes and strongly inhibit growth [13,16,33,34]. Yet interactions among constituents can be synergistic or antagonistic. Extracts rich in eugenol, estragole, and methyl eugenol were as effective as Chinese cinnamon extracts that lacked these compounds, and similar activity has been observed in oils with differing thymol and carvacrol proportions [15,17]. In this study, estragole was one of the few constituents present across all six oils, alongside very low amounts of ρ-cymene and terpinene-4-ol. The three oils with the highest estragole, basil then laurel then anise, were also the most effective in preventive applications, which suggests a role for elicitation. Estragole-rich matrices have primed resistance in several species, mostly against fungal pathogens, and multiple essential oils have enhanced host defenses *in planta*, although validation for *Actinidia*-*Psa* is still needed [12,44,45,46,47,48]. Under our experimental conditions, a purely surface protective effect seems less likely, given the high volatility of most constituents and the 14-day interval between application and inoculation. However, less volatile EO components, such as fatty acids, sterols and waxes, may persist for longer and could be retained by the surfactant used for emulsification [22,49].

Cardamom and laurel were more effective when applied after infection (as a curative approach), with cardamom showing limited preventive effects. Although their dominant constituents differ, β-himachalene in cardamom and 1,8-cineole in fennel, both have recognized antibacterial activities, and the overall activity could also reflect interactions among minor constituents [21,39,50]. In contrast, laurel and cumin reduced *in planta* bacterial density in both timing windows. For cumin, this dual action may derive from a diversified profile rather than a single driver. Cumin contained five constituents above 5%, with cuminaldehyde as the major component. Cuminaldehyde inhibits biofilm formation and proteolysis, reduces virulence, and increase membranes permeability in bacteria, with cumin oils rich in 3-caren-10-al and cuminaldehyde showing strong antioxidant capacity that could support plant defenses [51,52,53,54]. Similarly, cinnamaldehyde, the dominant component of *Cinnamomum cassia* oil, also targets bacterial membranes, and it has also been implicated in elicitation in apple leaves [15,55,56,57]; however, whether cumin also elicits defenses in *Actinidia* remains to be tested. Laurel and anise, both dominated by anethole, behaved similarly *in vitro* against *Psa*7286 and as preventive treatments, consistent with the antioxidant properties of anethole that may aid host defenses [58]. Nevertheless, their protective behavior diverged: laurel exhibited strong curative activity while anise did not, reflecting differences in their chemical profiles. For example, laurel contained higher levels of estragole plus fenchone and D-limonene, which were absent in anise. Although these compounds typically exhibit limited activity against Gram-negative bacteria, estragole has documented bactericidal effects on *Psa* and likely contributed to laurel’s superior curative efficacy [15,59,60,61,62,63].

Taken together, these results highlight that: (1) essential oil efficacy is timing dependent *in planta*, so preventive and curative windows should be tested explicitly; (2) composition alone does not dictate performance because interactions among constituents are common; selecting a single molecule based only on relative abundance is risky, and (3) cumin and laurel emerge as robust candidates against both *Psa* e and *Pfm*, while basil and anise appear most useful preventively and cardamom and fennel curatively. Future work should map strain level response surfaces, quantify elicitation markers in *Actinidia* following oil application, assess formulation variables that improve persistence without phytotoxicity, and test rational combinations at subinhibitory doses to exploit synergy while meeting practical constraints in kiwifruit orchards.

## 4. Materials and Methods

### 4.1. Essential Oil Emulsions

Plant essential oils from anise (*Pimpinella anisum* L., Ref.: AT155), basil (*Ocimum basilicum* L., Ref.: AT324), cardamom (*Elettaria cardamomum* L. Maton, Ref.: AT056), cumin (*Cuminum cyminum* L., Ref.: AT057), fennel (*Foeniculum vulgare* Mill., Ref.: AT111) and laurel (*Laurus nobilis* L., Ref.: AT116) were purchased from H. Reynaud & Fils, Montbrun Les Bains, France. For each oil, a 90% (*v*/*v*) stock emulsion was prepared by mixing the pure, undiluted oil with sterile distilled water containing 2% (*v*/*v*) Tween 20 as emulsifier. To control for surfactant effects, matching Tween 20 solutions without essential oil were prepared at the corresponding final concentrations and used as non-treated controls. All emulsions were shaken for 30 s immediately before being used to ensure homogeneity.

### 4.2. Bacterial Strains and In Vitro Antibacterial Assay

Two *Psa* strains, 7286 (CFBP, Italy) and 1F (ANSES, France), and two *Pfm* strains, 18,804 and 19,441 (both ICMP, New Zealand and Australia, respectively), were used [2,64]. Along the text, strains are referred to as *Psa*7286, *Psa*1F, *Pfm*18804 and *Pfm*19441. Cultures were maintained on nutrient sucrose agar (NSA) at 27 °C in the dark. For inoculum, a single colony of each strain was grown overnight in liquid Luria–Bertani medium at 27 °C and 75 rpm.

Antibacterial activity was assessed on NSA plates by paper disk diffusion [65]. Bacterial suspensions were adjusted to OD_600_ = 1.0, approximately 1 × 10^9^ cells.mL^−1^, and spread onto separate plates. Sterile blank antimicrobial susceptibility disks (Thermo Fisher Scientific, MA, USA) were loaded with 10 µL of each essential oil emulsion or the corresponding Tween 20 control and placed at the center of inoculated plates. Plates were incubated at 27 °C for 48 h in the dark. Zones of growth inhibition were measured as the diameter of the inhibition halo, in millimeters, and compared with the negative controls. Each treatment was tested with three biological replicates, each comprising three technical replicates.

### 4.3. In Planta Validation of Antibacterial Activity Against Psa

Micropropagated *Actinidia chinensis* var. *deliciosa* ‘Tomuri’ plants, each with a single shoot 5 to 6 cm tall and 8 to 12 leaves, were obtained from QualityPlant (Castelo Branco, Portugal). Plants were maintained *in vitro* on modified full-strength Murashige and Skoog agar medium in a climate chamber as previously described [4].

Plants received essential oil treatments either 14 days before inoculation (preventive) or 14 days after inoculation (curative), as schematically depicted in Figure 2A. For each timing, nine plants per essential oil were treated. An additional set of nine plants per timing received 2% Tween 20 without essential oil as non-treated controls, resulting in a total of 126 plants. Treatments consisted of dipping shoots for 15 s in 0.1% *v*/*v* essential oil emulsions prepared from the stocks in Section 2.1. This concentration was selected based on preliminary tests that revealed phytotoxicity at higher levels.

For inoculation, a fresh suspension of *Psa*7286 at 1–2 × 10^7^ CFU·mL^−1^ in sterile Ringer’s solution was prepared on the day of challenge, which involved immersing plant shots in the inoculum for 15 s. After treatment in the preventive schedule or after inoculation in the curative schedule, plants were maintained for 28 days in a climate chamber (Fitoclima 5000 EH, Aralab, Rio de Mouro, Portugal) with a 16 h light photoperiod and a light intensity of 200 μmol.s^−1^.m^−2^, 22 °C during the light period and 20 °C during the dark period. Sampling occurred at 14 days post inoculation in the preventive experiment and at 28 days post inoculation in the curative experiment. For this, plants were removed from the growing media, and shoots were homogenized in Ringer’s solution for endophytic *Psa* quantification by serial dilution and plating [4]. For each condition, three biological replicates were analyzed, each obtained by random pooling of three shoots.

### 4.4. Chemical Characterization by GC–MS

Essential oils were diluted in 10% ethanol (GC grade, Merck Group, Darmstadt, Germany). Samples were analyzed in triplicate on a Varian CP 3800 gas chromatograph with autosampler coupled to a Varian Saturn 4000 ion trap mass spectrometer, controlled by Varian software version 6.9.1. Separation used a VF 5 ms capillary column, 30 m × 0.25 mm × 0.25 µm, with high-purity helium as carrier at 1.0 mL.min^−1^ in splitless mode. The oven program was 40 °C for 1 min, ramp 5 °C min^−1^ to 250 °C with a 5 min hold, then 5 °C min^−1^ to 300 °C with no hold. Additional specifications followed Barros et al. [66]. Compounds were identified in a non-targeted approach by comparison with pure and mixed standards and by spectral matching against the NIST EPA NIH Mass Spectral Library version 2.2.

### 4.5. Statistical Analysis

Differences among means were evaluated by analysis of variance (ANOVA) followed by Fisher’s least significant difference test at *p* < 0.05. Analyses were performed in GraphPad Prism version 10.4.1, GraphPad Software, Boston, MA, USA.

## 5. Conclusions

This study extends prior *in vitro* observations for basil, cumin, fennel and laurel by demonstrating their activity against *Psa* and, for the first time, showing inhibitory effects against *Pfm*. It also documents previously unreported activity for anise and cardamom. Crucially, *in planta* experiments show that EOs can reduce endophytic *Psa* populations, but that *in vitro* efficacy does not translate directly to *in planta* effectiveness and is strongly dependent on application timing. The results identify distinct functional roles among the oils tested: basil and anise were effective only as preventive treatments, cardamom showed curative efficacy, and cumin and laurel performed well in both preventive and curative contexts. Chemical profiling linked bioactivity to candidate constituents such as anethole, estragole, himachalene, cuminaldehyde and 1,8-cineole, while emphasizing that overall composition alone does not reliably predict biological outcome because interactions among constituents are common. Future work should focus on standardizing EO formulations and doses for field use to reduce reliance on copper and antibiotics while minimizing phytotoxicity. Key priorities include benchmarking preventive and curative windows across diverse *Psa* and *Pfm* strains and *Actinidia* genotypes, testing rational EO combinations at subinhibitory concentrations to identify synergistic blends, and quantifying host elicitation markers and physiological responses (transient and cumulative) to clarify mechanisms of action. Together, these steps will help translate the present findings into robust, scalable tools for kiwifruit disease management.

## Figures and Tables

**Table 1:** Most abundant constituents of anise, basil, cardamom, cumin, fennel, laurel essential oils shown as relative composition percent from GC-MS analysis. Remaining identified compounds are listed in Supplementary Material. Abbreviations: ND—not detected.

Constituents	Anise	Basil	Cardamom	Cumin	Fennel	Laurel
Anethole	88.89	1.74	0.18	0.14	ND	78.02
3-Carene	ND	0.03	2.23	13.77	1.46	0.70
1,8-Cineole *	ND	3.19	0.14	3.39	68.71	ND
Cuminaldehyde	ND	ND	ND	47.95	ND	0.08
β-Cymene	ND	ND	1.20	18.67	1.18	1.10
Estragole	2.67	93.28	0.46	0.53	0.45	6.66
Fenchone	ND	0.10	ND	ND	0.03	9.08
β-Himachalene	0.37	ND	77.72	ND	17.15	0.07
D-Limonene	ND	0.20	7.42	0.40	2.84	1.84
Linalyl acetate	0.12	0.32	3.77	0.34	1.83	0.02
Longifolene	3.76	ND	ND	ND	ND	ND

* Also known as Eucalyptol.

## Data Availability

The original contributions presented in this study are included in the article/Supplementary Materials. Further inquiries can be directed to the corresponding author.

## Supplementary Materials

The following supporting information can be downloaded at: https://www.mdpi.com/article/10.3390/plants14243825/s1.
